# Preoperative easily misdiagnosed pure spinal epidural cavernous hemangioma: clinical-radiologic-pathologic correlations

**DOI:** 10.3389/fneur.2025.1615451

**Published:** 2025-09-02

**Authors:** Jingwen Li, Zhongyin Guo, Yang Liu, Mengqi Xu, Peng Wang, Feng Chen, Yuecheng Zeng, Peng Peng

**Affiliations:** ^1^Department of Oncology, Xiangyang Central Hospital, Affiliated Hospital of Hubei University of Arts and Science, Xiangyang, China; ^2^Department of Neurosurgery, Xiangyang Central Hospital, Affiliated Hospital of Hubei University of Arts and Science, Xiangyang, China; ^3^Institute of Neuroscience and Brain Diseases, Xiangyang Central Hospital, Affiliated Hospital of Hubei University of Arts and Science, Xiangyang, China

**Keywords:** cavernous hemangioma, epidural, diagnosis, treatment, spine

## Abstract

**Objective:**

Pure spinal epidural cavernous hemangiomas (PSECHs) are exceedingly rare vascular anomalies, often underreported and prone to misdiagnosis. This study aims to synthesize existing literature alongside seven cases from our institution to elucidate the clinical, imaging, and pathological characteristics of PSECHs for improving the clinical diagnosis and treatment of this disease.

**Methods:**

The clinical data of published literature and seven patients diagnosed with PSECHs admitted to the Department of Neurosurgery of Xiangyang Central Hospital from January 2013 to November 2022 were retrospectively analyzed for pre- and post-operative imaging findings, clinical manifestations, treatment history, pathologic characteristics, and treatment effects.

**Results:**

Approximately a hundred cases of PSECHs had been documented in the literature, with a clinical misdiagnosis rate reaching 91.3%. Among the seven patients studied, five were female, with a mean age of 49.4 years. In five cases, the lesions were located in the thoracic vertebral segment, while the cervical and lumbar vertebral segments each accounted for one case. One patient presented exclusively with radicular symptoms, four exhibited solely spinal cord symptoms, and two experienced both spinal cord and radicular symptoms. Two cases were initially misdiagnosed as meningiomas prior to surgery (2/7, 28.6%), with one case not accurately identified during the operation, leading to an erroneous aspiration as surgical blood seepage. Alongside the preoperative diagnosis of vascular lesion in case 7, we experienced a 42.9% (3/7, 42.9%) rate of preoperative misdiagnosis. Six lesions (6/7, 85.7%) demonstrated isointensity on T1-weighted images and hyperintensity on T2-weighted images, with all lesions exhibiting homogeneously strong enhancement. The “double tail” sign and fusiform shape in the sagittal view were observed in six lesions. Complete resection was achieved in all patients, resulting in excellent clinical outcomes. No symptoms or lesions recurred during the follow-up period.

**Conclusion:**

PSECHs are rare vascular malformations and easily misdiagnosed preoperatively. Accurate identification of the imaging characteristics of PSECHs is crucial for their diagnosis and subsequent management. Surgical total excision remains an effective treatment modality, and early intervention is recommended to prevent acute hemorrhagic events, which could adversely impact patient outcomes.

## Introduction

Cavernous hemangiomas, in general, are rare benign vascular tumors of the central nervous system and may manifest throughout the nervous system, with a predilection for the cerebral cortex, cerebellum, and spinal tissue (1). Within the spine, these lesions are predominantly located in the vertebral body, constituting 5–12% of all spinal vascular pathologies, followed by occurrences in the intramedullary or extramedullary subdural spaces (2). PSECHs are particularly rare, with only approximately one hundred cases documented in the literature. The rate of preoperative misdiagnosis has been reported to be as high as 91.3% (2) (Table 1), with the literature indicating misdiagnosis rates of 78.57% (3), 88.89% (4), and 66.67% (5) in various studies. Inaccurate preoperative diagnoses can lead to various complications, such as misidentifying a tumor as blood seepage, resulting in its non-detection during surgery. Consequently, this study retrospectively analyzed published literature and the clinical data of seven patients with PSECHs in the Xiangyang Central Hospital Neurosurgery Department from January 2013 to November 2022, in order to enhance clinicians’ understanding of the disease and decrease preoperative misdiagnosis rates.

**Table 1:** Basic characteristics of included studies (*n* = 55).

Included studies	Sex/age	Lesion site	Pre-operation MR characteristics			
			T1WI	T2WI	Enhancement	Misdiagnosis percentage of pre-operation (%)
Primary spinal epidural cavernous hemangioma: clinical features and surgical outcome in 14 cases	M/79	T6-7	Iso	Hyper	HE/Ob	78.57 (11/14)
	M/56	T2-4	Iso	Hyper	HE/Ob	
’1	M/42	T7-8	Hypo	Hyper	HE/Ob	
	M/15	C6-T2	Iso	Hypo	None	
	M/35	T2-4	Hyper	Hypo	Marginal	
	F/68	L2-3	Hypo	Hyper	HE/Ob	
1	M/66	T2-4	Iso	Hyper	HE/Ob	
	M/67	T3-4	Iso	Hyper	HE/Ob	
	M/24	C6-7	Hyper	Hypo	None	
	F/35	C4-5	Iso	Hyper	HE/Ob	
	M/67	T2-3	Iso	Hyper	HE/Ob	
	F/54	C7-T1	Iso	Hyper	HE/Ob	
	F/65	T12-L2	Hypo	Hyper	None	
	F/50	T4-5	Iso	Iso	HE/Ob	
Clinical features and long-term surgical outcomes of pure spinal epidural cavernous hemangioma—report of 23 cases	M/48	L4	Hypo	Hyper	HO/Ob	91.30 (21/23)
	F/45	S1–2	Iso	Hyper	HO/Ob	
	F/52	L4–5	Iso	Hyper	HO/Ob	
	M/56	T6	Iso	Slight Hyper	HO/Ob	
	F/46	C7-T1	Iso	Hyper	HO/Ob	
	M/53	T3–4	Iso	Hyper	HO/Ob	
	M/44	L3–4	Iso	Hyper	HO/Ob	
	M/38	L5	Hypo	Hyper	HO/Not Ob	
	F/55	T11–12	Iso	Hyper	HO/Ob	
	M/41	T2–4	Iso	Slight Hyper	HE/Ob	
	F/49	T9–11	Slight hype	Slight Hyper	HE/Ob	
	M/64	T9–11	Slight Hyper	Hyper	HO/Ob	
	F/70	T3–4	Iso	Hyper	HE/Ob	
	F/42	T4–7	Slight	Hyper	HO/Ob	
	M/59	T12–L1	Iso	Hyper	HE/Ob	
	F/56	L2–3	Iso	Hyper	HO/Ob	
	M/58	T2–3	Iso	Hyper	HO/Ob	
	F/54	L1–2	Slight	Slight Hyper	HE/Ob	
	F/64	T8–10	Iso	Hyper	HO/Ob	
	F/46	T3–9	Slight Hyper	Hyper	HE/Ob	
	F/50	T5–7	Slight Hyper	Hyper	HE/Ob	
	M/56	T5–6	Iso	Hyper	HO/Ob	
	M/38	T2–4	Iso	Hyper	HO/Ob	
Spinal dumbbell-shaped epidural cavernous hemangioma (CM): report of nine surgical cases and literature review	M/55	T2–4	Hypo	Hype	HO/Ob	88.89 (8/9)
	M/58	T3–4	Iso	Hyper	HO/Ob	
	M/79	T7–8	Iso	Hyper	HE/Ob	
	M/66	T2–3	Iso	Hyper	HO/Ob	
	M/63	T3–4	Iso	Hyper	HO/Ob	
	F/54	C7-T1	Iso	Hyper	HO/Ob	
	M/44	T7–9	Iso	Hyper	HO/Ob	
	F/69	T5–6	Iso	Hyper	HE/Not Ob	
	F/34	C3–4	Iso	Hyper	HE/Ob	
Spinal Epidural Cavernous Hemangiomas: A Clinical Series of 9 Cases and Literature Review	M/52	T3-T4	Iso	Mild Hyper	HO/Ob	66.67 (6/9)
	M/73	C7-T1	Iso	Hetero	HE/Not Ob	
	F/70	T12-L2	Iso	Hyper	HE/Not Ob	
	F/41	T5-T6	Iso	Hyper	HO/Ob	
	F/63	T10-T12	Iso	Hyper	HE/Ob	
	F/60	C6-T1	Iso	Hyper	HO/Ob	
	M/47	L4-L5	Iso	Hyper	HE/Not Ob	
	M/78	T7-T9	Iso	Hyper	HO/Ob	
	M/61	T6-T9	Iso	Hyper	HO/Not Ob	

Iso, isointense; Hyper, hyperintense; Hypo, hypointense; Hetero, heterogeneous; HE, heterogeneous; HO, homogeneous; Ob, obvious; CM, cavernous malformation.

## Materials and methods

### General information

This retrospective clinical investigation compiled comprehensive clinical data from patients treated for PSECHs at our institution during the specified period. The cohort comprised seven patients, including two males (28.6%) and five females (71.4%), with a mean age of 49.4 years, ranging from 43 to 54 years.

All patients exhibited a gradual onset of symptoms, primarily presenting with sensory and motor disturbances in the lower limbs, alongside neurological manifestations such as radiculopathy. Specifically, three cases presented with radiculopathy symptoms, including lower limb pain, toe numbness, and dizziness, while six cases exhibited varying degrees of spinal cord symptoms, such as limb weakness and numbness. Numbness in both lower limbs was observed in five cases, three of which also experienced decreased muscle strength in the lower limbs. Limb weakness was noted in four cases, radicular neuropathy in one case, and unilateral limb numbness in two cases. Regarding symptom progression, six cases demonstrated a gradual worsening of symptoms, one case experienced fluctuating symptoms, and none of the patients exhibited acute progression. The duration of symptoms ranged from 6 to 24 months, with a progressive worsening over time. Among the seven patients, five cases (71.4%) occurred in the thoracic segment, while one case (14.3%) each occurred in the lumbar and cervical segments. After discharge, all patients reported no neurological symptoms during outpatient visits or telephone follow-ups. Detailed clinical information is presented in Table 2.

**Table 2:** Detailed clinical information of seven cases.

Number	Lesion level	T1WI	T2WI	Enhancement	Shape in Sagittal position	Double tail sign	Intervertebral foramen extension	Vertebra destruction	Pre-ope diagnosis	Pre-ope Frankel grade	Degree of excision	Post-ope Frankel grade	Last-fol Frankel grade	Time from surgery to last-fol (months)	Recurrence
Case 1	T7-T9	Iso	Slight hyper	Homo	Fusiform	Yes	Yes	No	CM	D	Total	D	E	92	No
Case 2	T9-T11	Iso	Slight hyper	Homo	Fusiform	Yes	No	No	Meningioma	D	Total	D	D	135	No
Case 3	T10-T12	Iso	Slight hyper	Homo	Fusiform	Yes	No	No	CM	E	Total	E	E	19	No
Case 4	T6-T8	Iso	Hyper	Homo	Fusiform	Yes	Yes	No	CM	D	Total	D	D	80	No
Case 5	T7-T9	Iso	Slight hyper	Homo	Fusiform	Yes	Yes	No	CM	C	Total	D	D	105	No
Case 6	L1-L2	Iso	Slight hyper	Homo	Fusiform	Yes	No	No	Meningioma	E	Total	E	E	132	No
Case 7	C1-C2	Slight hyper	Hypo	Homo	Beaded	No	No	No	Vascular lesions	E	Total	E	E	77	No

Iso, isointense; Homo, homogeneous; Hyper, hyperintense; CM, cavernous malformation.

### Typical cases

#### Case 1

**Figure 1:**
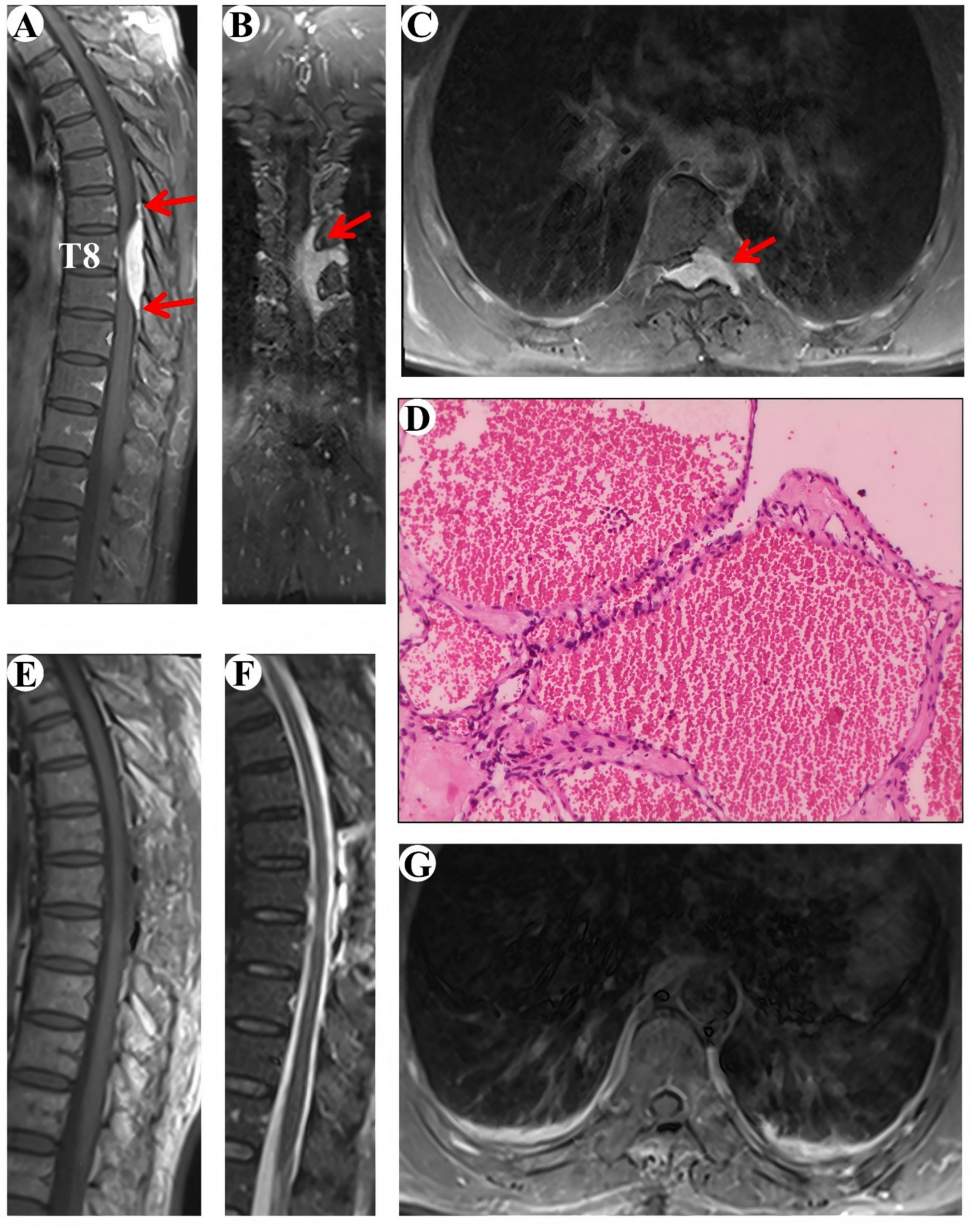
Clinical data pertaining to PSECH of the T7 and T9 vertebrae in Case 1. Typical “double tail” sign was marked with red arrows.

A 52-year-old male patient was admitted to the hospital due to an eight-month history of progressive numbness in his left toe, which had deteriorated and was now accompanied by bilateral lower limb weakness for the preceding 3 months. Sensory perception, both deep and superficial, was diminished below the T8 level in the chest and abdomen, as well as in the lower limbs. Muscle strength in the lower extremities was compromised, with a grading of 2–3 on the right side and 3 on the left. The MRI showed a subdural tumor lesion in the spinal canal between the T7 and T9 levels. The lesion, suspected to be a meningioma or cavernous hemangioma, measures about 3 cm by 5.5 cm and exhibited isointensity on T1-WI and hyperintensity on T2-WI, with obvious homogeneous enhancement and a characteristic “double tail” sign (Figures 1A–C). Surgical resection of the intraspinal mass was conducted via a posterior median approach under general anesthesia. Intraoperatively, a red, extra-dural soft tissue mass with a somewhat firm texture and a rich blood supply was identified, measuring approximately 1.5 cm x 1.0 cm x 1.0 cm, which differed from the typical light-yellow appearance of extra-dural fat. Part of the tissue extended into the left intervertebral foramina, showing a well-defined boundary and minor adhesion to the extra-dural membrane and nerve roots. Notably, there was an absence of a prominent drainage vein and arterial blood supply artery. Postoperative pathological analysis identified a cavernous vascular malformation (refer to Figure 1D). Follow-up images verified the full removal of the lesion and relief of spinal cord compression (Figures 1E–G). During a 92-month follow-up period, the patient exhibited grade V muscle strength in both lower limbs, and the numbness previously experienced in these areas had resolved.

#### Case 2

**Figure 2:**
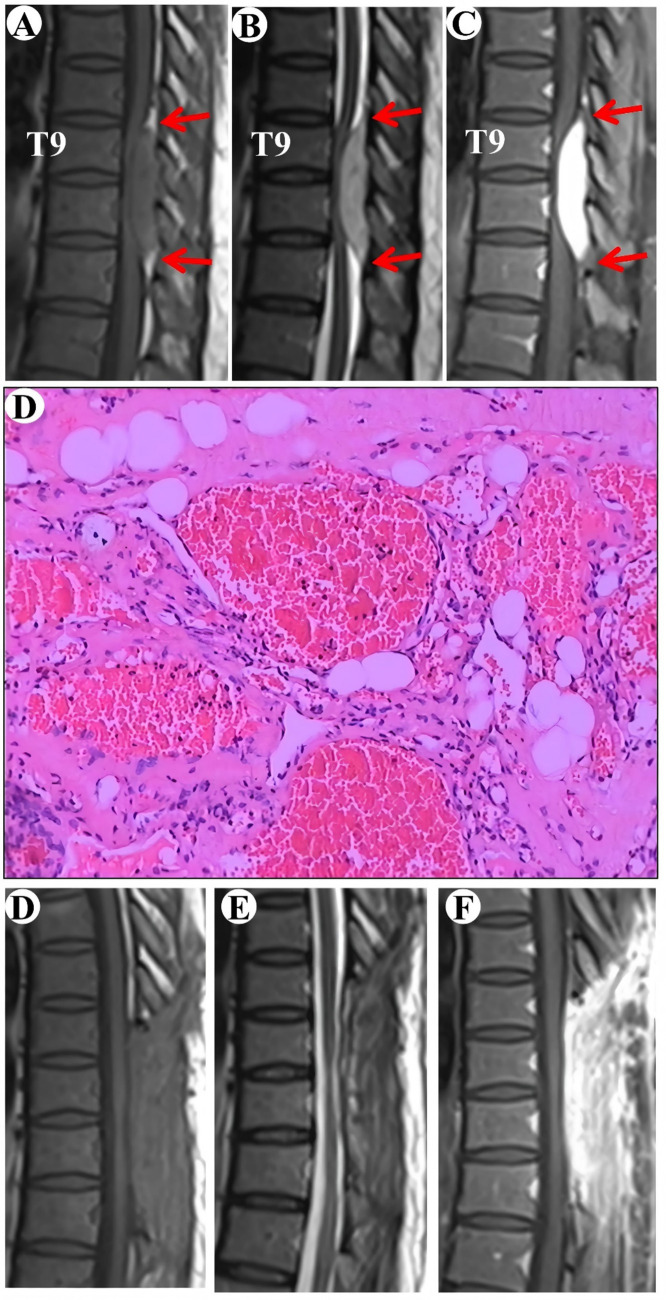
Clinical data pertaining to PSECH of the T9 and T11 vertebrae in Case 2. Typical “double tail” sign was marked with red arrows.

A 57-year-old female patient was admitted to the hospital with a two-year history of progressively worsening weakness in both lower limbs. Sensory perception, both deep and superficial, was reduced in the chest and abdomen below the T11 level. Muscle strength in the lower extremities was compromised, with a grade of 2 on the right side and 3 on the left. The MRI revealed a subdural tumor lesion in the spinal canal, extending from the T9 to T11 levels. The lesion, suspected to be a meningioma, measures approximately 1.5 cm by 5.5 cm and showed isointensity on T1-WI and hyperintensity on T2-WI, with obvious homogeneous enhancement and a typical “double tail” sign (Figures 2A–C). Surgical resection of the intraspinal mass was conducted via a posterior median approach under general anesthesia. A red, extra-dural soft tissue with a somewhat firm texture and a rich blood supply was identified during surgery, measuring around 1.5 cm x 5.5 cm, unlike the typical extra-dural fat, which is typically light yellow in color. Postoperative pathological analysis identified a cavernous vascular malformation (refer to Figure 2D). Follow-up images verified the full removal of the lesion and relief of spinal cord compression (Figures 2E–G). During a 135-month follow-up period, the patient exhibited grade V muscle strength in both lower limbs, and the numbness previously experienced in these areas had resolved.

#### Case 3

**Figure 3:**
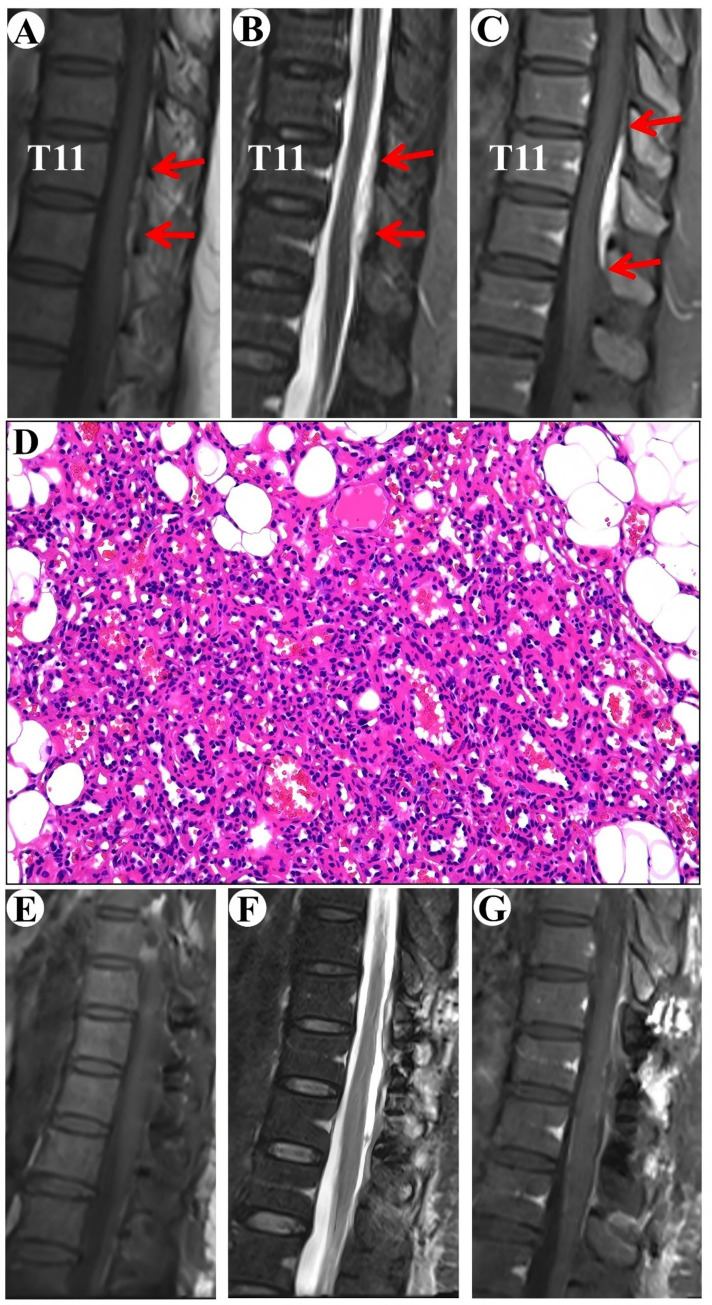
Clinical data pertaining to PSECH of the T11 and T12 vertebrae in Case 3. Typical “double tail” sign was marked with red arrows.

A 54-year-old female patient was admitted to the hospital with a one-year history of intermittent numbness in the right heel. The MRI indicated an epidural lesion within the T10-T12 spinal canal, suggestive of a possible cavernous hemangioma measuring approximately 1.5 cm by 4 cm. The lesion exhibited isointensity on T1-WI and hyperintensity on T2-WI, with obvious homogeneous enhancement and a typical “double tail” sign (Figures 3A–C).

Surgical resection of the intraspinal mass was conducted via a posterior median approach under general anesthesia. A red, extra-dural soft tissue with a rich blood supply was identified during surgery, measuring around 1.5 cm × 4.0 cm × 0.5 cm, with a distinct boundary and minor adhesion to the extra-dural membrane and nerve roots. Notably, there was an absence of a prominent drainage vein and arterial blood supply artery. Postoperative pathological analysis identified a cavernous vascular malformation (Figure 3D). Follow-up images verified the full removal of the lesion and relief of spinal cord compression (Figures 3E–G). During a 19-month follow-up period, the numbness in the right heel vanished.

#### Case 4

The patient, a 52-year-old woman, was admitted to the hospital due to numbness and weakness in both lower limbs for 2 years. Sensory perception, both deep and superficial, was reduced below the navel. Additionally, muscle strength in the both lower extremities had weakened, with a grading of 3.

**Figure 4:**
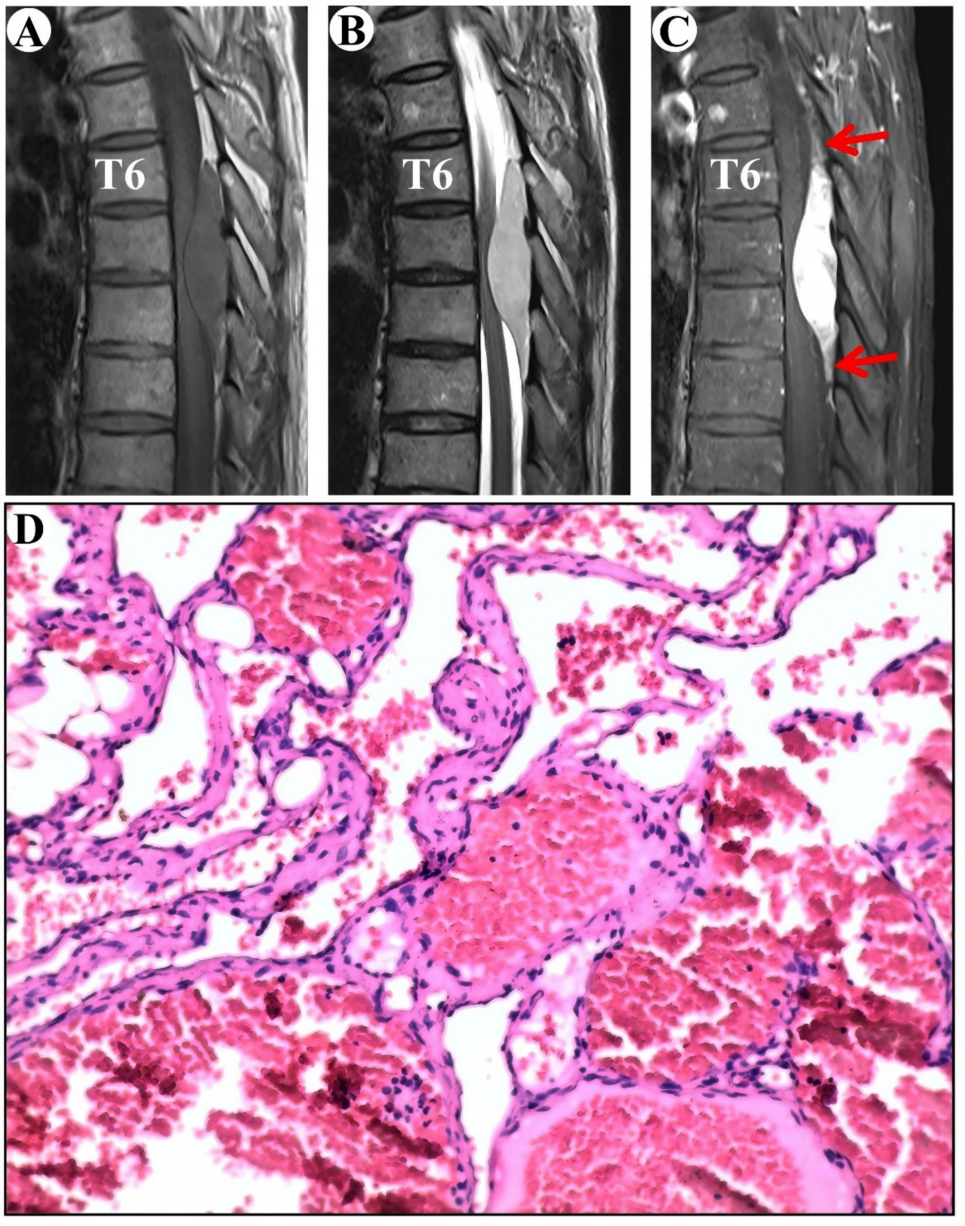
Clinical data pertaining to PSECH of the T6 and T8 vertebrae in Case 4. Typical “double tail” sign was marked with red arrows.

The MRI showed an epidural lesion in the spinal canal between the T6 and T8 levels. The lesion, suspected to be a cavernous hemangioma, measures about 1.9 cm by 8.1 cm and shows isointensity on T1-WI and hyperintensity on T2-WI, with obvious homogeneous enhancement and a typical “double tail” sign (Figures 4A–C).

Surgical resection of the intraspinal mass was conducted via a posterior median approach under general anesthesia. A red, extra-dural soft tissue with a rich blood supply was identified during surgery, with a distinct boundary and minor adhesion to the extra-dural membrane and nerve roots. Notably, there was an absence of a prominent drainage vein and arterial blood supply artery. Postoperative pathological analysis identified a cavernous vascular malformation (Figure 4D). During an 80-month follow-up period, the numbness below the navel vanished.

## Discussion

**Figure 5:**
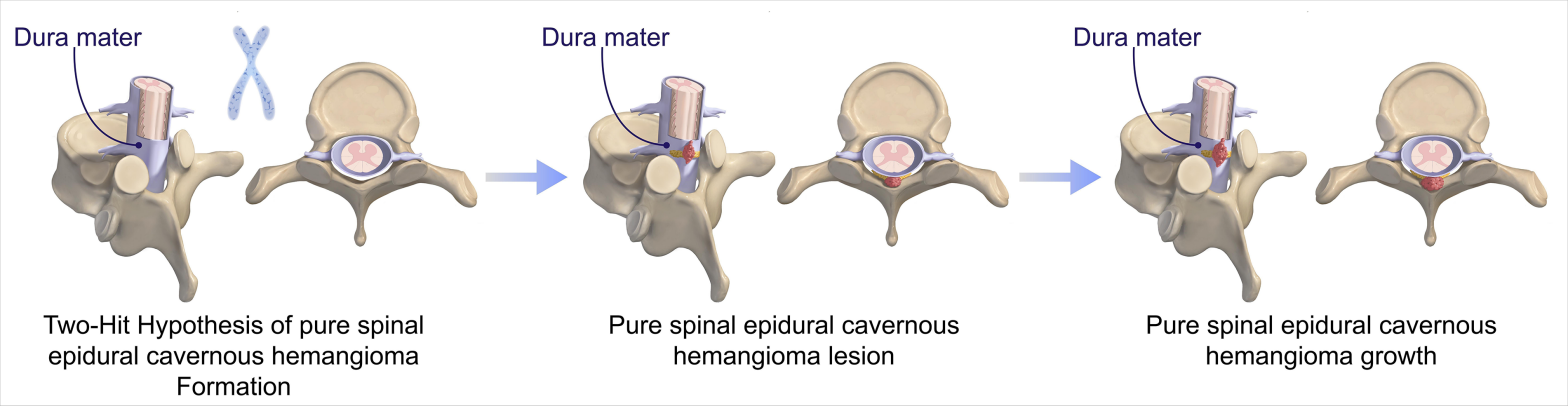
Progression of PSECHs pathogenesis: Two-Hit Hypothesis. The pathogenesis of PSECHs begins with an inherited or somatic mutation, followed by somatic mutations resulting in lesion genesis and growth.

Cavernous hemangioma is a type of vascular malformation characterized by an abnormal aggregation of thin-walled blood vessels, which lack both elastic fiber layers and muscular layers (1). Histopathologically, these lesions are marked by vessel dilation, reduced contact between endothelial cells lining the vessels, and a deficiency in junctional complex proteins (1). Cavernous hemangiomas may occur sporadically or be inherited, with the latter associated with loss-of-function mutations in the KRIT1/CCM1, CCM2, or PDCD10/CCM3 genes (1, 6). The inheritance pattern is autosomal dominant, where a “second hit” mutation in the other functional allele precipitates disease onset (1) (Figure 5). Cerebral angiography typically does not reveal any large feeding arteries or draining veins. These hemangiomas are predominantly intracranial and infrequently occur within the spinal canal, with an annual incidence of 2.2 per 10 million, accounting for 5 to 12% of intraspinal vascular conditions (1). Based on their anatomical location, cavernous hemangiomas are classified into intramedullary, subdural extramedullary, epidural extramedullary, and vertebral body types (2, 5, 7, 8). Among these, the intramedullary type is the most prevalent, whereas the pure epidural type is relatively rare, representing only 4% of all neuraxial epidural tumors. Pagni et al. indicated that the onset of the disease typically occurred between the ages of 30 and 60 (9). The thoracic segment was the most frequently affected area, accounting for 54 to 60% of cases, whereas the cervical segment constituted 30%, and the lumbar segment comprised 10%. In our study, the cohort consisted of five female and two male patients, with a mean age of 49.4 years. Among these patients, five lesions were located in the thoracic spine, and one each in the cervical and lumbar spines, consistent with findings reported in the literature.

The clinical manifestations of this disease are non-specific and predominantly involve symptoms of spinal cord compression at the affected segment, such as numbness, weakness, and paraplegia in the limbs below the affected segment. The initial presentation of the disease varies in severity and is generally categorized into three forms (10–13): (1) A slow onset, characterized by initial radicular pain that gradually progresses to numbness in the limbs and sphincter dysfunction. All cases in our study exhibited this form, and due to the lack of distinctive clinical features, achieving a definitive diagnosis prior to surgery is challenging. (2) An intermittent onset, which may involve repeated minor bleeding or intratumoral thrombosis, leading to intermittent and recurrent symptoms. (3) Stroke onset, also referred to as acute spinal cord apoplexy was frequently caused by the rupture of malformed blood vessels due to exertion, strenuous activity, or childbirth. This resulted in a short-term hemorrhage and the formation of an intramedullary hematoma, leading to rapid disease progression and severe neurological impairment. The optimal timing for surgical intervention in such cases remained a topic of debate. Several researchers advocated for early surgical removal of the hematoma and malformed blood vessels to alleviate nerve compression symptoms and maximize the restoration of spinal cord function (4, 14–16). However, during the acute stage of hemorrhage, particularly in emergency surgeries, the distinction between normal tissues and hemangiomas was often unclear, which can lead to spinal cord injuries and adverse outcomes due to limited visibility. Therefore, it is advised to address dehydration initially and perform surgery after symptoms have been alleviated or stabilized to achieve optimal results. In our study cohort, all patients exhibited symptoms that developed gradually and were resolved following surgical treatment.

MRI is the primary modality for the imaging diagnosis of this disease; however, the literature indicated a high preoperative MRI misdiagnosis rate of 91.3% (2) (see Table 1). The authors retrospective analyzed patients from 2001 to 2018, the time was earlier than our series. Secondly, 3 T-MRI was applied regularly in our center. Moreover, we routinely conduct preoperative multidisciplinary discussions, which mainly consist of neurosurgeons, radiologists, and pathologists. These points were the reasons of our lower misdiagnosis rate. Three cases of our series were initially misdiagnosed as meningiomas prior to surgery (3/7, 42.9%), with one case not accurately identified during the operation, leading to an erroneous aspiration as surgical blood seepage. Inaccurate preoperative diagnoses could lead to various complications, such as misidentifying a tumor as blood seepage, resulting in its non-detection during surgery. The MRI characteristics of this disease include uniform isointense or hypointense signals on T1-weighted imaging (T1WI) and hyperintense signals on T2-weighted imaging (T2WI) (2, 5, 11, 12, 17). Occasionally, mixed T1WI and T2WI signals are observed due to factors such as rupture and hemorrhage of cavernous hemangiomas, hematoma organization following repeated bleeding, fibrosis, or peritumoral hemosiderin deposition, accompanied by homogeneous enhancement. The lesion typically affects multiple segments, presenting as a fusiform or oval mass aligned with the longitudinal axis of the spinal cord. It is well-demarcated from the surrounding tissue and exhibits a “double tail” sign on the sagittal T1 enhancement sequence. Within our group, the enhanced MRI demonstrated uniform enhancement, a well-defined border with adjacent areas, appearing like a fusiform mass aligned with the spinal cord’s longitudinal axis, along with typical “double tail” sign.

Differentiating the disease from neurogenic tumors, lymphomas, metastases, and meningiomas in the epidural space is essential. ① Neurogenic tumors are generally round and exhibit hypointense signals on T1-weighted imaging (T1WI), while appearing slightly hyperintense on T2-weighted imaging (T2WI). Some neurogenic tumors may undergo cystic degeneration and necrosis, resulting in heterogeneous signal intensity (17). Lymphomas typically present as isointense on T1WI and hyperintense on T2WI, with the potential for moderate to significant enhancement following contrast administration. This condition is often accompanied by other systemic manifestations, complicating differentiation from cavernous hemangiomas, which can be definitively identified through pathological examination post-surgery (11). Metastatic tumors are usually associated with swelling of adjacent soft tissues and bone destruction, characterized by hypointense signals on T1WI and hyperintense signals on T2WI. Metastases often display ring-like enhancements and rarely show the uniform enhancement characteristic of cavernous hemangiomas, typically originating from primary tumor sites (11). Meningiomas are primarily located in the extramedullary subdural space and generally exhibit isointense signals on both T1WI and T2WI (17). Upon enhancement, the lesion exhibits a distinct uniformity, albeit to a lesser extent than that observed in cavernous hemangiomas. Enhancement of the adjacent dura is frequently observed, often presenting a “meningeal tail sign,” whereas cavernous hemangiomas typically exhibit a “double tail sign.”

Histopathologically, the lesions are composed of mature vessels with thin walls lined by endothelium, interspersed with loose connective and adipose tissue (1). The vessel walls are primarily lined by a single layer of flattened endothelial cells within collagenous tissue, lacking elastic and muscular tissue or neuronal elements. Thrombosis and remnants of previous hemorrhages are also evident in cavernous hemangiomas, particularly in larger lesions.

Surgical excision is the preferred treatment modality for this condition. The fundamental surgical objective is to excise as much of the lesion as possible while minimizing the risk of spinal cord damage. This condition is characterized by the absence of large supplying arteries or draining veins and is predominantly located on the dorsal aspect of the epidural space. Consequently, complete resection of the hemangioma is recommended (1). Intraoperative neuroelectrophysiological monitoring, including motor evoked potentials (MEP) and somatosensory evoked potentials (SSEP), was routinely employed in our center. Typically, the PSECHs were mild with dural or nerve root adhesions and exhibited a relatively distinct interface, allowing for precise sharp anatomical separation during surgical procedures. These points were added in the discussion section. The application of low-power bipolar coagulation on the tumor surface can effectively reduce the size of the cavernous hemangioma, thereby facilitating its complete resection. Cavernous hemangiomas are often easily separable from the dura or spinal nerve roots; however, their separation becomes more challenging when they invade the intervertebral spaces. Postoperative radiotherapy serves as an effective adjunct for tumors that are not fully excised. Nonetheless, stereotactic radiotherapy may be considered, given that traditional radiotherapy is not widely employed due to the potential risk of spinal cord radiation damage (12). In this cohort, all patients underwent complete intraoperative excision without the need for postoperative radiotherapy.

Based on the assessment of patients’ recovery status before and after surgery, it is evident that the preoperative neurological function significantly influences postoperative recovery outcomes. Patients exhibiting favorable preoperative neurological function, primarily characterized by sensory symptoms, tend to experience positive postoperative recovery. Conversely, those with compromised preoperative neurological status, notably indicated by reduced muscle strength, demonstrate slower postoperative recovery. Hence, it is imperative to address cavernous hemangioma within the spinal canal promptly, as early surgical intervention prior to the onset of irreversible neurological damage can lead to optimal surgical outcomes.

## Data Availability

The original contributions presented in the study are included in the article/supplementary material, further inquiries can be directed to the corresponding authors.
